# Sexual and reproductive health communication intervention for caretakers of adolescents: a quasi-experimental study in Unguja- Zanzibar

**DOI:** 10.1186/s12978-019-0756-z

**Published:** 2019-06-28

**Authors:** Saada A. Seif, Thecla W. Kohi, Candida S. Moshiro

**Affiliations:** 1 Department of Nursing and Public Health, School of Nursing, The University of Dodoma, P.O. Box 259, Dodoma, Tanzania; 2 Department of Nursing Management, School of Nursing, Muhimbili University of Health and Allied Sciences, P. O. Box 65001, Dar es Salaam, Tanzania; 3 Department of Epidemiology and Biostatistics, School of Public health and Social Sciences, Muhimbili University of Health and Allied Sciences, P. O. Box 65001, Dar es Salaam, Tanzania

**Keywords:** Adolescents, Caretakers, Sexual and reproductive health, Communication, Parents, Intervention effect, IMB-model

## Abstract

**Background:**

Caretakers/parents or parents figure need to be trained to promote effective communication about sexual and reproductive health to their adolescents. This study assessed the effect of an intervention aiming to improve caretaker-adolescent communication on sexual and reproductive health matters through improving information, motivation, and behavioral skills related to sexual health communication. The study also evaluated the relationship of information, motivation, and behavioral skills model-constructs with communication practice. Information-Motivation-Behavioural skills model was used as a framework to guide the intervention implementation and evaluation process.

**Method:**

This is a quasi-experimental non-randomized controlled pre- and post-test study which involved one thousand caretakers of adolescents in all the six districts of Unguja-Zanzibar. All participants completed interviewer-administered structured pre-test questionnaire. The experimental group then received sexual health communication intervention addressing the information, motivation, and behavioral skills related to sexual health communication, while the control group received the sexual health information only. All participants were then reassessed for their information, motivation, behavioral skills and their sexual health communication after 1 month, 6 months and at 1 year following the intervention. To evaluate the effect of intervention at the post-test measures, Univariate Analyses of Covariance was performed whereby the pre-test score and variables on which the groups differed were considered as covariates. Standardized mean difference statistics of Cohen’s d was used to calculate the effect size, and the cut-off point for the level of significance was set at two-sided, *p*-value < 0.05.

**Results:**

Results shows that the immediate post-test sexual health communication, motivation and behavioral skills scores were statistically significantly higher in the experimental group compared to control group (*p* < 0.05). Moreover, sexual health communication score after 6 months and at 1 year were statistically significantly higher in the experimental group compared to control group (*p* < 0.05). Information construct however did not differ between groups in post-test measures. Furthermore, results revealed that communication practice is statistically significantly associated with information, motivation and behavioural skills in post-test measures.

**Conclusion:**

The findings provided preliminary evidence for the effectiveness of SRH communication intervention and supported the significance of IMB model-constructs to inform the SRH-communication intervention and to guide the intervention evaluation.

## Plain-english summary

This study mainly focused on adolescents’ Sexual and Reproductive Health (SRH). With the expected impact of reducing risky sexual behaviour of adolescents, the strategy of utilizing parent or parent figure (caretakers) was adopted in which parenting practice of communicating with adolescents on sexual and reproductive health issues was targeted. Realizing the needs, strength and deficit of caretakers in relation to SRH communication, the intervention guided by the behavioral model (Information-Motivation-Behavioral skills (IMB) model) was implemented to equip caretakers with necessary SRH knowledge, motivation and skills to communicate. The effect of this intervention on improving SRH related knowledge, motivation, and skills was then evaluated after 1 month, and its effect on improving parent-child SRH communication was evaluated after 6 months and 1 year following the intervention.

Of the 836 respondents, 667 were female, 736 were married, 341 were of higher educational attainment. The average age of all participants was 45.7 years, and 350 have female adolescents and 640 live with their biological adolescents. The intervention resulted in increase of SRH related knowledge, motivation and skills to communicate after 1 month, and a sustained increase of parent-child communication after 6 months and 1 year follow up.

In conclusion: The study has found out that caretakers can adopt the role of SRH educators if they are provided with necessary support even in areas where cultural norms discourage such communication. IMB-based SRH communication intervention should be considered the panacea to empower the community so that parents could teach their adolescent children about SRH.

## Background

Sexual and Reproductive Health (SRH) becomes a major area of concern during adolescence because of the apparent risky sexual behaviours which include early age sexual debut, multiple sexual partners, unprotected sexual intercourse, and sexual intercourse while under the influence of alcohol or drugs [1, 2]. These behaviours increase the risk of unintended pregnancy and/or Sexually Transmitted Infections (STIs) including Human Immunodeficiency Virus (HIV) infection. In Tanzania more than half of the population is under the age of 20. About 13% of women would have had sex by the age of 15 years, and 59% by the age of 18 years [3]. HIV infection among young people is of particular concern whereby 1.4 million people are living with HIV and 5.1% prevalence is among 15 to 49-year-olds [4].

Communication about SRH between parents/caretakers and children/adolescents has received a great deal of attention recently. Evidences show that children who talk with their parents about sexual matters are more likely to postpone sexual activity, have fewer sexual partners and are more likely to use contraceptives and condoms [5–8]. Most caretakers however do not communicate with their adolescents because they find this task as daunting and they often feel ill-equipped. Moreover, caretakers do not communicate because of the belief that such communication is immoral, contrary to traditional values, and it is likely to encourage premarital sexual activity [9, 10]. Thus studies have called for the intervention involving parents, and it has been suggested that issues interrelated to sex and parental responsiveness should be addressed more systematically, as this may impact parent-child SRH communication practice [11–13].

Although studies on caretaker-adolescent communication on SRH are increasing in Sub Saharan Africa (SSA) [11], this area has not been well studied in Tanzania. The qualitative studies in Tanzania indicate that some parent-child communication does occur but not in a friendly way. Parents seem to be using fear, threats and physical discipline to ensure their adolescents do not engage in sexual activities. Communication takes the form of warning, and sometimes the language and expressions are ambiguous. Some topics, like the use of condoms are avoided and that communication is mainly about abstinence, HIV/AIDS and unwanted pregnancy [14, 15].

Zanzibar has been recognised for its rich and splendid cultural and religious legacy, far different from Tanzania Mainland, whereby any explicit discussion related to sexuality is normally discouraged. Therefore, in order to improve parent-child SRH communication in Zanzibar, there is a need to implement and evaluate the effectiveness of SRH communication intervention among caretakers of adolescents. In Zanzibar, the only SRH communication intervention implemented is DARAJA curriculum through UJANA project which utilised personnel from UMATI-Zanzibar branch [16]. The evaluation results of this project were not published. Moreover, the availability of training personnel from UMATI who were trained for this curriculum in Zanzibar has prompted us to replicate this intervention, and evaluate its effectiveness. In addition, it is important to note that the contents of DARAJA curriculum include interesting components necessary for behaviour change like SRH knowledge, motivation and skills. Therefore, this time we decided to incorporate the Information-Motivation-Behavioural skills (IMB) model as a framework to guide the implementation and evaluation of this intervention. The use of this model will help us to take into account the specific cultural and religious context of Zanzibar during the intervention implementation. The use of this model in this research also was expected to strengthen its significance in informing SRH communication intervention projects and guide their evaluation. The main objective of this study therefore was to evaluate the effect of SRH communication intervention in improving caretaker-adolescent communication through improving the information, motivation and behavioural skills among caretakers of adolescents in Unguja-Zanzibar.

In this study, Berlo’s Sender-Message-Channel-Receiver (SMCR) model of communication [17] was applied to describe the communication process, and Information-Motivation-Behavioural skills (IMB) model [18] was applied to describe the occurrence of communication practice of caretakers. Berlo’s SMCR model of communication focuses on encoding and decoding processes which happen before sender sends the message and before receiver receives the message respectively. It has mainly four components to describe the communication process. They are sender, message, channel and receiver. The model describes several factors affecting the individual components in the communication making the communication more efficient. The factors that are related to sender and receiver are communication skills, attitude, knowledge, social system and culture. According to this model, if the sender and receiver have good communication skills, the message will be communicated better and the receiver can grasp the message. The attitude of the sender and the receiver creates the effect of the message, also familiarity with the subject of the message makes the communicated message have its effect more. Values, beliefs, laws, rules, religion and many other social factors affect the sender’s way of communicating the message and cultural differences make messages different and thus my hinder effective communication [17].

Information-Motivation-Behavioural Skills (IMB) model [19] was adopted to inform the SRH communication intervention and guide its evaluation. Theory-based research is necessary to identify the determinants of SRH communication which can be targeted in intervention. The IMB model was first used on HIV preventive behaviour change to examine risk reduction behaviours in at-risk adolescents [18, 20, 21]. In more recent years, the model has been used to predict more general health-related behaviours. The concept of this model has been empirically validated to a diverse population including adults and adolescents and diverse health behaviours [19, 22–24]. This model has received considerable attention because it does not only provide a relatively simple explanation for complex health behaviours but also identifies constructs that are needed for behaviour change [25]. When using this model, it is recommended that one should begin with elicitation research to identify the deficits and strengths in relation to IMB components. The next step is to develop intervention components to address the deficits, and finally make the evaluation to determine the effectiveness of the intervention.

**Fig. 1:**
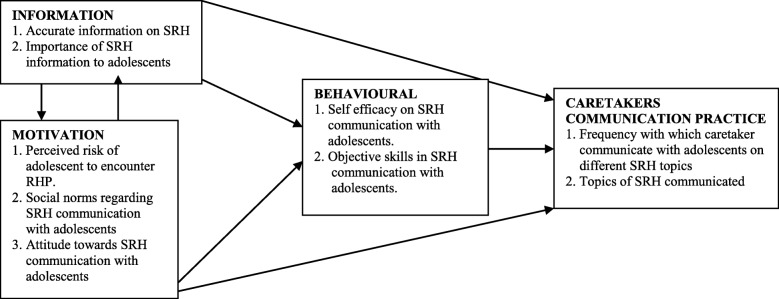
The Information-Motivation-Behavioural Skills (IMB) Model Source: (Fischer & Fisher, 1992)

According to the IMB model (Fig. 1), information (knowledge), motivation and behavioural skills are the fundamental determinants for the initiation and maintenance of health behaviours. It postulates that performing a health behaviour is a function of the extent to which someone is well-informed about the behaviour, motivated to perform the behaviour (e.g., has positive personal beliefs and attitudes towards the behaviour, perceiving vulnerability and have social support to practice the behaviour), and has the behavioural skills (objective skills) requisite skills to execute the behaviour and confidence (perceived efficacy) in their ability to do so across various situations [26].

**Fig. 2:**
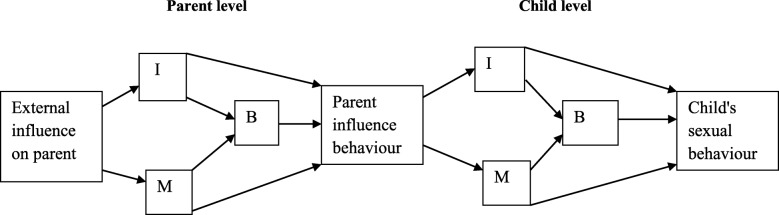
IMB-model Parent expansion [Adopted from Theory of Planned Behaviour Parent Expansion (Katherine Hutchinson & Wood, 2007)]

The IMB model can be extended to evaluate the impact of distal factors that influence an individual’s behavior (Fig. 2). Distal factors, such as parents’ influence on adolescent’s behaviors, are important to measure. Thus, one can never view individuals’ behavior without taking account the environment and relationships. The parent expansion of IMB model is adopted from the parent expansion of Theory of Planned Behaviour (TPB) [27].

In this study, we therefore hypothesized a direct effect from SRH communication intervention to each of the mediating variables (Information, Motivation and Behavioral skills). We also hypothesized a direct effect from each of the mediating variables to the outcome variable which is SRH communication. We then hypothesized a direct effect from the SRH communication intervention to the outcome SRH communication. This study therefore is set out to test the hypothesis that “The mean-score of SRH communication, information, motivation and behavioural skills at post-test measure of the experimental group is not equal to that of the control group adjusting for their pre-test mean-scores and the measures on which the groups differed”.

## Methodology

### Setting and design

This study was conducted in Unguja-Zanzibar, a part of United Republic of Tanzania. The design of this study was quasi-experimental non-randomized controlled pre and post-test research design. The study involved all the 6 districts within the Island whereby two wards (one experimental and one control) in each district were selected. In total, there were six experimental wards and six control wards. The distance between experimental and control wards was considered so as to avoid the likelihoods of intervention diffusion between wards [28]. Although there are minor differences between the experimental and control wards due to their distance apart, the groups have much in common in terms of their tradition and social structure since they are located in the same district.

To carry out this research, three specific phases were undertaken as follows: First, the baseline phase aimed to determine the existing level of information, motivation and behaviour skills pertaining to communication on SRH, and this constituted the pre-test scores. The second phase which was carried out at least 1 month after the first phase involved the implementation of SRH communication intervention [DARAJA (THE BRIDGE) curriculum] to experimental groups. The control groups did not receive any of the experimental intervention but were exposed to SRH information only. Thirdly, evaluation was carried out at three time-intervals; one-month after completion of the intervention programme, followed by 6 months after the intervention, and then at 1 year follow up, participants were assessed to determine if the intervention has had sustained effect on their communication practice. All surveys were administered using the same structured questionnaire.

### Study participants

The study population were all male and female caretakers of adolescents aged 15–19 years. Study subjects were eligible to participate in the study after given their consent; these caretakers were either biological parents or parent figures who must have stayed continuously with the adolescents for at least 2 years prior to the survey. Caretakers who were staying with young people who were married were considered ineligible for the study. Moreover, participants do not have to be literate to participate in this intervention study.

A three-stage probability sampling technique was used to select the individuals. In all the six districts in Unguja, two out of eight wards in each district were purposively selected, one being experimental and the other being control, making a total of 12 wards. Simple random sampling was then used to select 3 streets (shehia) in each ward making a total of 36 shehias. Systematic random sampling was then used to select 28 (1000 participants/36 shehias) households from a sampling frame consisting of approximately 450 houses in each shehia [29]. After the first household entered, the next 16th (450/28) household with eligible subjects was chosen for interviewing. This process continued until the target sample size was obtained. In each household, if both male and female caretakers were present, a male caretaker was deliberately chosen because of prior experience that male caretakers are difficult to reach because they have their activities mostly outside their home, unlike female caretakers [30]. In houses with multiple households, (for example compound houses) one household was randomly selected for interviewing.

**Fig. 3:**
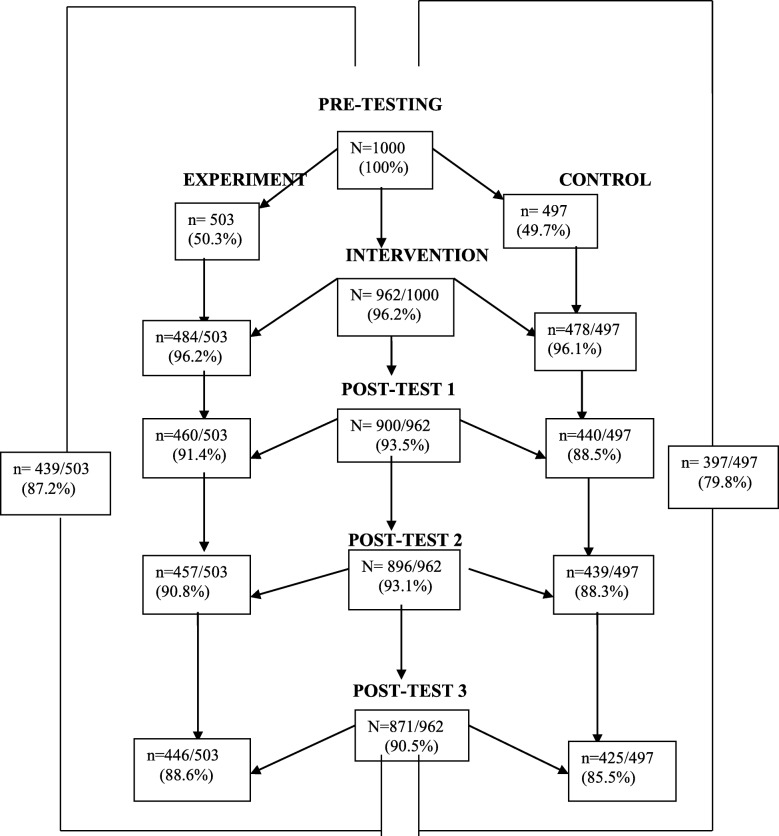
Flow Diagram

The number of participants involved in each time-point of the study is provided in flow chart of follow up (Fig. 3). One thousand participants completed a pre-test assessment at baseline with 503 participants forming the experimental group, and 497 participants forming the control group. Out of 1000 participants, 962 (96.2%) participated in phase two of the study with 484 (50.3%) participants in the experimental group. At one-year follow-up, data was available from 871/1000 of the baseline cohort.

To be included in the analysis of intervention effect, participants had to have attended all the intervention sessions and completed the pre-test and all the post-tests measures. In all, 836/1000 were included in the final analysis with 439/503 (87.2%) participants of the experimental group and 397/497 (79.8%) of the control group. Most of the participants who had dropped out were not present at their homes during the data collection exercise, and two had died. A comparison of people included in the analyses with those excluded because of loss to follow-up revealed no significant differences in terms of demographic characteristics and baseline outcome measures.

### Intervention procedure

This research protocol was approved by the Muhimbili University of Health and Allied Sciences (MUHAS), and by the Ministry of Health and Social Welfare in Zanzibar. Written informed consent was obtained from all interested caretakers.

The DARAJA / BRIDGE curriculum intervention employed in this study was developed by the American Red Cross Society and adapted for Tanzania by the collaboration of Tanzania Red Cross and Family Health International (360) Tanzania. In this study, the contents of DARAJA curriculum were related to IMB constructs such that the Information construct was dealt in lesson 4 and 6, Motivation construct was dealt in lesson 1,2 and 3 and Behavioral skills was dealt in lesson 5 and 7. This intervention is delivered in three phases (Bridges); Bridge 1 engages caretakers alone, Bridge 2 involves adolescents alone, and Bridge 3 involves both adolescents and caretakers combined together. Therefore, according to this curriculum structure, the intervention is carried out for two conservative days, in which in the first day bridge 1 and bridge 2 were implemented separately, and on the second day bridge 3 was implemented. In each bridge, the training sessions lasted for 5 h and 25 min. The intervention delivery methods included lectures, games, group discussions, role-plays and brainstorming. On the other hand, the SRH information-only workshops was delivered in the same way as for the experimental groups by using lecture method and discussion.

Initially, the elicitation research as recommended by Fischer and Fisher [18] employed qualitative Focus Group Discussion (FGD) and a quantitative questionnaire with a sample of a target population was carried out [31–33]. While most parts of the intervention contents based on the available DARAJA curriculum were maintained, the interview and FGD of the initial phase helped to better understand important components that needed to be emphasised during the second phase, so as to fit the context of caretakers in Unguja. For example, during FGDs, discussing the use of condoms and other contraceptive methods with adolescents was strongly opposed by participants; this part had to be omitted in adolescents’ sessions. However in caretakers sessions the topic on the use of condoms was introduced as one of the safest family planning methods, with the belief that if it was accepted they would later on acknowledge its significance in preventing pregnancy even to adolescents.

### Measures

A standardized scale has not yet been developed to measure the components of the IMB model for caretaker-adolescent’s SRH communication. Questions were developed from the literature on adolescents and sex education to measure sexual communication. An IMB measure used in another study [34] on behavioral change was used in the present study and guided the development of information, motivation and behavioral skills scale to measure SRH communication practice. Content validity of this scale was assessed through peer review and internal consistency reliability for each measure was calculated through a pilot study on 50 caretakers. After revisions, the final version of the scale was prepared. It took 10 min to complete the questionnaire and it was administered by trained interviewers in Kiswahili language spoken by all participants.

*Information construct* assessed one’s knowledge of SRH by 15 items. Participants were required to mention spontaneously the contents and importance of SRH (e.g. what topics of SRH a caretaker discussed with the adolescent? One point was awarded for a correct-match item mentioned. The maximum score of the information construct was 15 points, (Cronbach’s Alpha coefficient was 0.42 at pre-test and 0.93 at post-test).

*Motivation* assessed caretakers perceived risk (3 items), social norms (3 items) and attitude (5 items) towards SRH communication to adolescents (e.g., Communicating SRH matters with adolescents will promote promiscuity). Items on a Likert scale with options ranging from 1 = strongly disagree to 4 = strongly agree, and the maximum score possible for this construct was 44. Cronbach’s alpha coefficient was 0.68 at pre-test and 0.89 at post-test.

*Behavioral skills* assessed perceived self efficacy (4 items) and perceived objective skills (4 items) of communicating with adolescents. Items on a Likert scale ranging from 1 = very hard to 4 = very easy, and from 1 = very ineffective to 4 = very effective (e.g., I can describe my ability to talk to my adolescent as very ineffective or very effective). Maximum score possible for this construct was 32. Cronbach’s alpha coefficient was 0.83 at pre-test and 0.88 at post-test. *Caretakers-adolescents communication on SRH* was assessed using two measures; global communication measure and the detailed examination of communication on specific sexual topics (overall measure of communication). Global measures (2 items) assessed if caretakers had ever communicated with their adolescent and if they have done so in the past 30 days. The response options was 1 = never to 4 = a lot. The overall communication was estimated using a weighted measure of family sexual communication scale (7 items) [34]. In the present study, the instrument asks respondents to indicate on a Likert the extent to whether seven specific sexual topics have been discussed to either female or male adolescents, (abstinence, pregnancy, safer sex, HIV/STIs, contraceptives use, abortion, and homosexuality). Example of the question was: How frequently do you talk to your adolescent female/male about pregnancy? Scores are computed by summing all items, with higher scores indicating greater amounts of sexual communication between parents and adolescent. The maximum score is 28 point, Cronbach’s alpha was 0.81 at pre-test and 0.93 at post-test.

### Data analysis

Analysis were performed with SPSS Version 22. Chi-square test was applied to compare demographic characteristics between experimental and control groups. Bivariate correlation between IMB variables and communication practice was calculated and Pearson correlation coefficients were reported.

To evaluate the effect of intervention at the posttest measures of IMB constructs and communication practice, two models of univariate analyses of covariance (ANCOVA) were performed. The first one was for examining the immediate effect of intervention (after 1 month) on IMB constructs and communication practice measures. The second model was for the long-term effect of intervention (at 6 months and 1 year follow-up) on communication practice measure. The pretest score of IMB constructs and communication practice measures and measures on which the groups differed were considered as covariate variables. Adjusted means scores for IMB constructs and communication practice were presented. Standardized mean difference statistics of Cohen’s d was used to calculate the effect size, and the cut-off point for level of significance was set at a two-sided *p*-value < 0.05.

Our analysis based on complete cases analysis or list-wise deletion whereby all cases with missing data were omitted. List-wise deletion is known to produce unbiased estimates and conservative results if the assumption of MCAR (Missing Complete At Random) is satisfied [35], and this was confirmed by comparing dataset with missing value and the other containing no missing value using t-test. The results revealed no significant differences in the sample between the two data sets. Moreover, none of the demographic characteristics and baseline outcome measures were significantly different between the follow-up sample and those who were lost to follow-up.

## Results

The final analysis included 836 participants with 439/503 (87.2%) of the experimental group and 397/497 (79.8%) of the control group Demographic data show that the sample of caretakers was predominantly female 667 (79.8%), married 736 (88.4%), of higher educational attainment 341 (40.8%), and with mean age of 45.7 years (SD = 10.9). Majority of respondents have female adolescents 350 (41.9%), and majority stay with their biological adolescents 640 (76.6%), while only 37(4.5%) stays with adolescents of other family members. The groups differed significantly on sex, age and education level (Table 1).

**Table 1:** Group Differences of Socio-Demographic Characteristics of Caretakers

Characteristics	Experimental (*n* = 439)		Control (*n* = 397)		χ^2^	p
	No.	%	No.	%		
Sex						
Male	71	16.2	98	24.7	9.37	.002
Female	368	83.8	299	75.3		
Age in years						
Mean (SD)	46.09	10.8	45.34	11.0	11.92	.008
35–39	157	35.8	165	41.6		
40–49	114	26.0	108	27.2		
50–59	114	26.0	65	16.4		
60+	54	12.3	59	14.9		
Marital status						
Single	7	1.6	6	1.5	2.87	.41
Married	381	86.8	358	90.2		
Divorced	12	2.7	6	1.5		
Widow/widower	39	8.9	27	6.8		
Education level						
No formal education	74	16.9	118	29.7	19.5	<.001
Primary education	170	38.7	133	33.5		
Secondary and higher education	195	44.4	146	36.8		
Occupation						
Farmer/fisherman	211	48.1	219	55.2	4.53	.21
Employed	43	9.8	37	9.3		
Petty business	111	25.3	87	21.9		
Housewife	74	16.9	54	13.6		
Caretaker’s adolescents’ sex						
Caretakers having male adolescents only	115	26.2	100	25.2	3.07	.22
Caretakers having female adolescents only	172	39.2	178	44.8		
Caretakers having both male and female adolescents	152	34.6	119	30.0		
Relationship with adolescent						
Biological child	343	78.1	297	74.8	1.75	.42
Child of other family member	20	4.6	17	4.3		
Both biological and of other family member	76	17.3	83	20.9		

### Immediate effect of intervention on SRH information, motivation, behavioral skills and communication practice

The estimated pre-test and 1 month post-test means scores from Univariate ANCOVA analysis for the immediate effects of intervention on SRH information, motivation and behavioral skills of SRH communication adjusted for pretest scores, age, sex and education level appears in Table 2.

**Table 2:** Effects of Intervention on Information, Motivation, Behavioural Skills and Communication at post tests (*N* = 836)

Measure	Experimental (*n* = 439)	Control (*n* = 397)	F- value	*p*-value	Effect size Cohen’s *d*
	Mean (SD)	Mean (SD)			
Information					
Pre-test	10.00 (2.1)	10.28 (1.8)	4.17	0.04	
Post-test	9.00 (4.8)	9.41 (5.5)	1.26	0.26	0.07
Motivation: Perceived risk					
Pre-test	5.58 (1.5)	5.23 (1.7)	10.02	0.002	
Post-test	6.62 (2.7)	5.92 (2.8)	4.58	< 0.001	0.3
Social norms					
Pre-test	7.64 (1.4)	7.71 (1.5)	0.56	0.46	
Post-test	8.32 (1.6)	8.25 (1.5)	0.46	0.51	0.05
Attitude					
Pre-test	13.44 (2.0)	13.27 (1.8)	1.51	0.22	
Post-test	12.52 (2.6)	11.61 (2.6)	26.6	< 0.001	0.3
Behavioural skills Self-efficacy					
Pre-test	8.73 (2.4)	8.04 (2.5)	17.12	< 0.001	
Post-test	11.60 (2.7)	11.41 (2.8)	0.95	0.33	0.07
Skills					
Pre-test	7.46 (2.6)	7.43 (2.5)	0.48	0.83	
Post-test	11.19 (2.9)	10.50 (3.1)	10.81	0.001	0.23
Communication					
Pre-test	16.26 (4.6)	15.05 (4.8)	13.98	< 0.001	
Post-test at 1 month follow up	22.46 (4.9)	20.99 (5.2)	16.74	< 0.001	0.3
Post-test at 6 month follow up	23.58 (4.5)	22.12 (5.2)	17.9	< 0.001	0.3
Post-test at 1 year follow up	24.15 (3.7)	21.83 (6.3)	40.44	< 0.001	0.4

#### Information

Participants in the experimental group did not significantly differed with the control group in reporting SRH information after the intervention F(1, 827) = 1.26, (*p* = 0.26).

#### Motivation

Post-test attitude F(1,827) = 49.4, (*p* < 0.001) and perceived risk F(1, 827) = 12.5, (*p* ≤ 0.001) were statistically significantly greater in the experimental group compared to the control group; with small effect size (d = 0.3) which indicates a non-overlap of 21.3% in the two distributions. However, social norms did not significantly differ by condition at post-test F(1, 827) = 0.46, (*p* = 0.51).

#### Behavioral skills

Post-test perceived skills was statistically significantly greater in the experimental group compared to the control group; F(1, 827) = 10.81, (*p* ≤ .001). Although the mean difference is highly statistically significant, effect size was small (d = 0.2) which indicates a non-overlap of 14.7% in the two distributions, suggesting the mean difference is not important practical. On the other hand, there is no enough evidence to support the difference observed of perceived efficacy score between the experimental group and the controls F(1, 827) = 0.95, (*p* = 0.33).

#### SRH communication

As can be shown in Table 2, post-test SRH communication was statistically significantly greater in the experimental group compared to the control group; F(1,827) = 16.74; (*p* ≤ 0.01) with small effect size (d = 0.3) which indicates a non-overlap of 21.3% in the two distributions.

### Long term effect of intervention on SRH communication

The estimated 6 months and 1 year follow-up post-tests mean scores from univariate ANCOVA analysis for the effects of intervention on SRH communication practice appear in Table 2. The analysis was adjusted for age, sex, education level, 1 month and 6 months measures of SRH communication (that is for 6 months follow-up and 1 year follow-up respectively).

#### Effect of intervention on SRH communication at 6 months follow up

6 months after the completion of the intervention participants were asked how often they discussed each of SRH topic to either female or male adolescents in the preceding 6 months. The results show that SRH communication was statistically significantly greater in the experimental group compared to the control group; F(1,827) = 17.9, (*p* < 0.001] with small effect size (d = 0.3) which indicates a non-overlap of 21.3% in the two distributions.

#### Effect of intervention on SRH communication at 1 year follow-up

1 year after the intervention, participants were asked how often they discussed each of SRH topic to either female or male adolescents in the preceding year. The results show that SRH communication was statistically significantly greater in the experimental group compared to the control group; F(1,827) = 40.44, (*p* < 0.001) with small effect size (d = 0.4) which indicates a non-overlap of 27.4% in the two distributions.

### Correlation between communication practice and information, motivation and behavioural skills at 1 year post intervention

Bivariate correlation was run to assess the relationship between communication practice and constructs of the IMB model. Preliminary analysis showed the relationship to be monotonic as assessed by visual inspection of a scatter plot. There was a significant positive correlation between communication and information [r = 0.22, *p* < 0.001], communication and motivation [r = 0.07, *p* = 0.05], and communication and behavioural skills [r = 0.43, *p* < 0.001].

### Path analysis

**Fig. 4:**
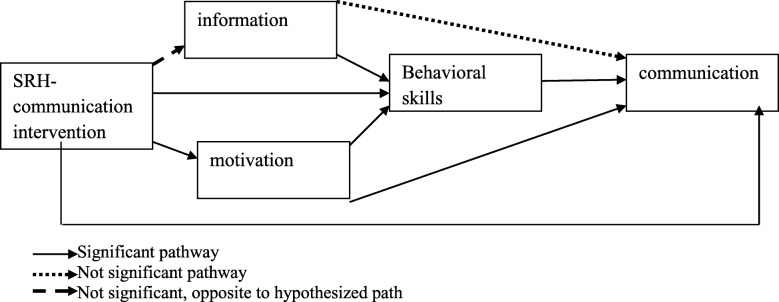
SRH-communication intervention study model Path analysis

As hypothesized by the study model, SRH communication intervention had significantt effect on Motivation and Behavioral skills but not in Information. Moreover, Motivation and Behavioral skills showed a direct significant effect on communication, while Motivation also showed a significant indirect effect through behavioral skills. Finally, SRH communication intervention showed a significant effect to the communication practice (Fig. 4).

## Discussion

This study assessed the effect of a conceptually based intervention focusing on enhancing parent-child communication on SRH matters through improving information, motivation, and behavioral skills among caretakers of adolescents. The study also aimed to evaluate the relationship of IMB model constructs with communication practice.

Findings showed that at baseline, sex, age and education level of the two groups were significantly related to SRH communication such that female caretakers, those of 50–59 years of age, and of higher education attainment were more likely to have had communicated with their adolescents. At the same time same sex communication was more common before the intervention. This could be due to feeling of shame and embarrassment when discussing SRH matters with the opposite sex [33]. Interestingly, after the intervention there was no significant difference in communication among caretakers of the experimental group within these variables.

Findings confirmed that caretakers who were exposed to intervention demonstrated significant improvement in motivation, behavioral skills and communication at immediate post-test, and significant sustained effects on communication practice after 6 months and after a one-year follow up than the control group. Despite the low non-overlap in the experimental and control distributions which may indicates it is not practical significant, the reported increase in SRH communication with adolescents over time suggests that the intervention may have helped overcome traditional and cultural barriers that restrict parent-child communication about SRH.

Traditionally, there were social structures responsible for preparing adolescents to adulthood, usually a member from the extended family of the same sex (e.g. aunt (Somo) for girls, and uncle for boys. They usually emphasize on hygiene for girls, and ones chaste was cherished more for girls than boys. However, direct conversation between parents and children about sex and sexual issues was a taboo [36]. These traditions have largely disintegrated with urban type of life and modernization, introduction of various sources of communication like the internet and western Television programs, thus people’s perceptions on traditional practices changed and take them as outdated. The findings therefore demonstrated that the intervention may have been successful in in influencing caretakers to play the non-traditional role of SRH educator, and it presented reasons to progress away from these restrictive taboos.

Despite the significant effect of intervention on motivation, behavioral skills and communication practice, there was no sufficient evidence to support the difference information-mean scores between experimental and control group at post test. This is not surprising given that most of SRH information provided to the experimental group was closely related to that which was given to the control group. Similar findings have been reported in the study of Cornman et al., (2007) [37] which utilized an IMB–model based intervention for inducing condom use behavior.

This study revealed that information, motivation, and behavioral skills have a significant positive correlation with communication practice. The evidence from literature suggests that accurate information about SRH, high levels of SRH communication motivation and strong SRH communication behavioral skills are associated with higher levels of SRH communication behavior [38]. Therefore these variables must be considered as key factors in designing SRH parent–child communication interventions. Despite the moderate association observed, identifying determinants of each component of IMB model to improve SRH communication among caretakers of adolescents may help to shape more effective interventions for promoting SRH communication among caretakers of adolescents.

Since behavioral skills construct demonstrated a high correlation with communication practice, and the best way to impose skills is through direct contact where participants could get a chance to practice the behaviour and get individualized feedback at the same time. Therefore, providing this intervention message via mass media is not advised though it is expected to reach higher number of people at lower cost, but will not be as effective as expected.

Community based SRH communication programmes are insufficient, but have promising results. Some intervention programmes implemented in SSA [39–41] targeted parents/adults focusing on increasing SRH communication with adolescents. Such studies have utilised similar elements of parental responsiveness including knowledge, motivation, comfort, skill and confidence as utilised in this study. Consistent with those interventions, a series of facilitated sessions were carried out and participants were followed over a period of time to determine the sustained effect of intervention on SRH communication practice. This was done because behaviour development occurs over time following several training sessions. There was congruence between this study and existing literature with regards to the methods of intervention-delivery used, which include role play, discussion, games and lectures. As for the studies done in SSA, these methods are believed to help the participants to develop communication skills and help them realise the importance of confronting cultural norms and taboos in protecting their children from reproductive health problems [39, 41, 42].

## Limitations

The current study findings have important theoretical and practical implications for refining parent-child SRH communication interventions; however, there are some limitations that are worth mentioning and taken into account in the interpretation of the findings.

One limitation of this study is that the intervention was held for only 2 days, this duration was not adequate for participants to process and practice the skills learned and to totally change the unhelpful cultural and religious norms that had been practiced for so many years. To overcome this, effort was made to ensure each participant had an opportunity to practice the skills more than once during the intervention. A series of six-three-hour sessions weekly-intervention is recommended as what was done in Family Matter Project-Tanzania whereby participants had more opportunity to practice at home the skills learned and then share the outcomes with their peers on their return [43].

Secondly, caretakers were asked retrospectively on their communication practice with their adolescents. Recall bias and social desirability could create response biases and may have lead to over or under reporting of communication practice. Future researches should consider to interview parent-child dyad so that the validity of parent’s response could be counterchecked and verified against child’s response.

Thirdly, more women participated in the intervention compared to men. This may have lead to overestimation of communication prevalence among the participants. Therefore caution should be taken when interpreting the results. Future studies should explore more about male perceptions towards SRH communication with adolescents since male involvement is as equally important as that of female in influencing adolescents’ risky sexual behaviours.

## Conclusion

The findings provided preliminary evidence for the effectiveness of SRH communication intervention and supported the significance of IMB model-constructs to inform the SRH-communication intervention and to guide the intervention evaluation. The study has found out that caretakers can adopt the role of SRH educators if they are provided with necessary support even in areas where cultural norms discourage such communication. IMB-based SRH communication intervention should be considered the panacea to empower the community so that parents could teach their adolescent children about SRH.

The practical implication of this study in informing future interventions is that community and family interactions shape individual behavior and are crucial to understand to better meet the population needs. Therefore one should look beyond the individual, both community and family level factors are salient in shaping caretakers’ cognitions about sexual health communication with their children. Furthermore; the provision of more time, follow-up session, and multisession-interventions are more likely to result in changes on social norms that have been practiced for many years and thus will improve SRH communication in a considerable amount. The theoretical implication of this study is that the development of parent-child communication on SRH issues has been highlighted in IMB-model which suggests that communication practice is related to the extent to which the person is informed, motivated and has behavioral skills to communicate. Therefore, and as suggested from the results, the study concludes that if caretaker perceives the risk, has supportive social norms, positive attitude, self-efficacy and communication skills, all these give him/her reason to communicate with adolescent about SRH issues. The success of DARAJA curriculum in Unguja holds promise for other intervention programmes in a range of settings with differing initial perceptions about discussing SRH with preadolescents.

## Data Availability

The datasets used and/or analysed during the current study available from the corresponding author on reasonable request.

## Abbreviations

AIDS: Acquired Immuno Deficiency Syndrome
ANCOVA: Univariate Analyses of Covariance
HIV: Human Immunodeficiency
IMB: Information Motivation Behavioural-skills
MCAR: Missing Complete At Random
PTB: Planned Theory of Behaviour change
RH: Reproductive Health
SMCR: Source, Message, Channel, Receiver
SPSS: Statistical Package for Social Sciences
SRH: Sexual and Reproductive Health
SSA: Sub Saharan Africa
STIs: Sexually Transmitted Infections
UMATI: Uzazi na Malezi bora Tanzania (A non-government organization dealing with reproductive and child health)

## References

[1] Babalola Stella, Tambashe B. Oleko, Vondrasek Claudia (2005). Parental Factors and Sexual Risk-Taking among Young People in Côte d'Ivoire. African Journal of Reproductive Health.

[2] Kirby D (2011). Reducing adolescent sexual risk: a theoretical guide for developing and adapting curriculum based programs: ETR associates.

[3] Commission ZA, unicef (2015). The Adolescent Experience In-Depth. ZAC and TACAIDS.

[4] TACAIDS T, Zanzibar A (2011). Commission (ZAC), National Bureau of Statistics (NBS), Office of the Chief Government Statistician (OCGS), and ICF International. 2013. Tanzania HIV/AIDS and Malaria Indicator Survey.

[5] Kirby D, Laris B, Rolleri L (2005). Impact of sex and HIV education programs on sexual behaviors of youth in developing and developed countries: family health international, YouthNet Program Durham, NC.

[6] Kirby D, Laris B, Rolleri L (2007). Sex and HIV education programs: their impact on sexual behaviors of young people throughout the world. J Adolesc Health.

[7] Rogers AA, Ha T, Stormshak EA, Dishion TJ (2015). Quality of parent–adolescent conversations about sex and adolescent sexual behavior: an observational study. J Adolesc Health.

[8] Wang Z (2009). Parent-adolescent communication and sexual risk-taking behaviours of adolescents.

[9] Organization WH (2007). Summaries of projects in developing countries assisting the parents of adolescents.

[10] Vandenhoudt H, Miller KS, Ochura J, Wyckoff SC, Obong'o CO, Otwoma NJ (2010). Evaluation of a US evidence-based parenting intervention in rural Western Kenya: from parents matter! To families matter!. AIDS Educ Prev.

[11] Bastien S, Kajula LJ, Muhwezi WW (2011). A review of studies of parent-child communication about sexuality and HIV/AIDS in sub-Saharan Africa. Reprod Health.

[12] Kamangu AA, John MR, Nyakoki SJ (2017). Barriers to parent-child communication on sexual and reproductive health issues in East Africa: a review of qualitative research in four countries. J Afr Stud Dev.

[13] Wamoyi J, Fenwick A, Urassa M, Zaba B, Stones W (2010). Parent-child communication about sexual and reproductive health in rural Tanzania: implications for young people's sexual health interventions. Reprod Health.

[14] Kajula L (2005). Cross-generation communication on sexuality in times of HIV/AIDS as perceived by adolescent girls and their parents in Dar Es Salaam, Tanzania.

[15] Kajula LJ, Sheon N, De Vries H, Kaaya SF, Aarø LE (2014). Dynamics of parent–adolescent communication on sexual health and HIV/AIDS in Tanzania. AIDS Behav.

[16] USAID. The UJANA Project, Tanzania Youth HIV Prevention Project. Cooperative Agreement No. 621-A-00-06-00010-00, Quarterly Report: April–June 2011. 2011. [Online]. Available at: pdf.usaid.gov/pdf_docs/pdacw490.pdf. Accessed 18 May 2016.

[17] Berlo DK (1960). The process of communication; an introduction to theory and practice.

[18] Fischer J, Fisher A (1992). Changing AIDS risk behaviour. Psychol Bull.

[19] Fisher JD, Fisher WA, Bryan AD, Misovich SJ (2002). Information-motivation-behavioral skills model-based HIV risk behavior change intervention for inner-city high school youth. Health Psychol.

[20] Fisher JD, Fisher WA (2000). Theoretical approaches to individual-level change in HIV risk behavior. Handbook of HIV prevention: Springer.

[21] Fisher WA, Fisher JD (1993). A general social psychological model for changing AIDS risk behavior.

[22] Fisher JD, Fisher WA, Misovich SJ, Kimble DL, Malloy TE (1996). Changing AIDS risk behavior: effects of an intervention emphasizing AIDS risk reduction information, motivation, and behavioral skills in a college student population. Health Psychol.

[23] Singh S (2003). Study of the effect of information, motivation and behavioural skills (IMB) intervention in changing AIDS risk behaviour in female university students. AIDS Care.

[24] Zarani F, Besharat MA, Sarami G, Sadeghian S (2012). An information–motivation–behavioral skills (IMB) model-based intervention for CABG patients. Int J Behav Med.

[25] Chang SJ, Choi S, Kim S-A, Song M (2014). Intervention strategies based on information-motivation-behavioral skills model for health behavior change: a systematic review. Asian Nurs Res.

[26] Fisher WA, Fisher JD, Harman J. The information-motivation-behavioral skills model: a general social psychological approach to understanding and promoting health behavior. Soc Psychol Found Health Illn. 2003;1:82–106.

[27] Katherine Hutchinson M, Wood EB (2007). Reconceptualizing adolescent sexual risk in a parent-based expansion of the theory of planned behavior. J Nurs Scholarsh.

[28] West SG, Biesanz JC, Pitts SC. Causal inference and generalization in field settings: experimental and quasi-experimental designs. Handb Res Methods Soc Pers Psychol. 2000;13:40–84.

[29] Office of Chief Government Statician, President’s Office, Finance, Economy and Development Planning (2010). Tanzania Demographic Health Survey.

[30] Ali S, Manongi R (2010). Caretakers acceptability in the provision of information on sexuality to adolescents using information motivation behavioral skills (IMB) model in urban district Zanzibar. East Afr J Public Health.

[31] Seif SA, Kohi TW (2014). Caretaker-adolescent communication on sexuality and reproductive health: my perceptions matter; a qualitative study on adolescents’ perspectives in Unguja-Zanzibar. Health.

[32] Seif SA, Kohi TW, Mselle LT (2016). Caretaker’s perceptions on caretaker-adolescent communication on sexual and reproductive health in Unguja-Zanzibar: implication for intervention. Health.

[33] Seif SA, Moshiro CS (2017). Caretaker-adolescent communication on sexual and reproductive health: a cross-sectional study in Unguja-Tanzania Zanzibar. BMC Public Health.

[34] Fisher TD (1987). Family communication and the sexual behavior and attitudes of college students. J Youth Adolesc.

[35] Kang H (2013). The prevention and handling of the missing data. Korean J Anesthesiol.

[36] Mlunde LB, Poudel KC, Sunguya BF, Mbwambo JK, Yasuoka J, Otsuka K (2012). A call for parental monitoring to improve condom use among secondary school students in Dar Es Salaam, Tanzania. BMC Public Health.

[37] Cornman DH, Schmiege SJ, Bryan A, Benziger TJ, Fisher JD (2007). An information-motivation-behavioral skills (IMB) model-based HIV prevention intervention for truck drivers in India. Soc Sci Med.

[38] Fishbein M, Bandura A, Triandis H, Kanfer F, Becker M, Middle stadt, SE (1991). Factors influencing behavior and behavior change final report–Theorist’s workshop.

[39] Bhana A, Petersen I, Mason A, Mahintsho Z, Bell C, Mckay M (2004). Children and youth at risk: adaptation and pilot study of the CHAMP (Amaqhawe) programme in South Africa. Afr J AIDS Res.

[40] Kiragu K, Watson C, Muhwezi M, Kibombo R, Nelson T (2007). Straight talk campaign in Uganda: parent survey.

[41] Phetla G, Busza J, Hargreaves JR, Pronyk PM, Kim JC, Morison LA, Watts C, Porter JD (2008). “They have opened our mouths”: increasing women's skills and motivation for sexual communication with young people in rural South Africa. AIDS Educ Prev.

[42] Lefkowitz Eva S., Stoppa Tara M. (2006). Positive sexual communication and socialization in the parent-adolescent context. New Directions for Child and Adolescent Development.

[43] Masanja P (2016). The impact of families matter project on parent-child communication: case study of Mbagala-Temeke District in Dar Es Salaam Tanzania. The Open University of Tanzania.

